# Patient characteristics associated with response to NSAID monotherapy in children with systemic juvenile idiopathic arthritis

**DOI:** 10.1186/s12969-017-0219-4

**Published:** 2018-01-05

**Authors:** Anjali Sura, Christopher Failing, Julie Sturza, Jasmine Stannard, Meredith Riebschleger

**Affiliations:** 1 University of Michigan, 1500 E Medical Center Dr, Ann Arbor, MI 48109 USA; 2 IHA Rheumatology Consultants, 4990 W Clark Rd, Suite 300, Ypsilanti, MI 48197 USA

**Keywords:** Systemic juvenile idiopathic arthritis (sJIA), Non-steroidal anti-inflammatory drugs (NSAIDs), NSAID monotherapy, Therapeutics, Risk stratification

## Abstract

**Background:**

Systemic juvenile idiopathic arthritis (sJIA) is an auto-inflammatory disease characterized by fever, arthritis, and ≥1 of rash, generalized lymphadenopathy, hepato/splenomegaly, and serositis. Non-steroidal anti-inflammatory drugs (NSAIDs) are among the initial treatments of sJIA, but there is currently no evidence indicating which children should undergo a trial of NSAID monotherapy and which should not. Our objective is to identify presentation characteristics which are associated with response and lack of response to a trial of NSAID monotherapy.

**Methods:**

This is a retrospective single-center cohort study of children diagnosed with sJIA from 2000 to 2014. Patient demographics and disease characteristics were investigated to identify predictors of response to NSAID monotherapy.

**Results:**

Eighty-seven children were newly diagnosed with sJIA 2000-2014. Thirteen of the 51 children who received NSAID monotherapy achieved clinically inactive disease (CID) without other medications. Age at presentation (≤8 years old), initial joint count (≤5), and C-reactive protein (CRP) (≤13 mg/dL) at diagnosis were associated with achievement of CID on NSAIDs alone. Physicians were less likely to trial NSAID monotherapy if the patient had either serositis or macrophage activation syndrome (MAS) at diagnosis. Ultimate achievement of CID and time to CID were not significantly affected by whether the patient received a trial of NSAID monotherapy.

**Conclusions:**

While a subset of children with sJIA can achieve CID with NSAID monotherapy, we recommend *against* a trial in patients who are >8 years old, with >5 joints involved, or with CRP > 13 mg/dL. Patients who undergo a trial of NSAID monotherapy should follow up within 2-4 weeks to evaluate for possible need for drug escalation. Clinical trials are necessary to confirm these findings.

## Background

Over the last 20 years, the outcomes of juvenile idiopathic arthritis (JIA) have markedly improved. Decreases in morbidity and mortality have occurred alongside increases in knowledge regarding the pathophysiology of JIA and the development of new medications that prevent long-term joint damage [1]. In the case of systemic JIA (sJIA), new agents have transformed treatment of the disease. In the past, many pediatric rheumatologists treated sJIA using a pyramid approach, starting with non-steroidal anti-inflammatory drugs (NSAIDs) and/or corticosteroids prior to initiating disease-modifying anti-rheumatic drugs (DMARDs) or biologic agents.

However, the Childhood Arthritis and Rheumatology Research Alliance (CARRA) Consensus Treatment Protocols (CTPs), published in 2012, does not include any arms with NSAID monotherapy [2]. This is contrary to the 2011 *ACR Recommendations for the Treatment of Juvenile Idiopathic Arthritis* and the 2013 update, which includes 4 options for the initial treatment of sJIA: NSAIDs, corticosteroids, DMARDs, and biologic agents [3]. Each of these drug classes has unique characteristics, such as potential adverse effects, mechanism of drug delivery (oral, subcutaneous, or infusion), and expected time to improvement. Drug class characteristics, child/caregiver preferences, and physician preferences contribute to the choice of a specific agent for a specific child. Unfortunately, the absence of comparative trials limits the ability to make evidence-based suggestions about the treatment of specific children, so recommendations to date have primarily relied on expert consensus. Based upon this expert consensus, the 2013 recommendations suggest that NSAID monotherapy be considered as a treatment option for children whose physician global rating of disease activity is less than 5 on a 10-point scale with any number of active joints. Given the emerging data indicating a window of opportunity in the early treatment of sJIA [4], transitioning to glucocorticoids, IL-1 blockade, or IL-6 blockade is recommended for any children who continue to have active disease after 1 month.

Identifying patient characteristics which are associated with a response to a trial of NSAID monotherapy can aid pediatric rheumatologists in choosing medications for the initial treatment of sJIA. This will help balance the risks and benefits of therapy for specific children. The objective of this cohort study is to review and analyze the treatment and outcomes of all children diagnosed with sJIA at a tertiary care center over a 15-year period with regard to treatment with NSAID monotherapy.

## Methods

### Subjects

After IRB exempt status was approved, an administrative data query was conducted and identified all children 0-18 years of age with a visit to the authors’ health system associated with the ICD-9 code for sJIA (714.30) between 1/1/2000 and 12/31/2014. As this ICD-9 code (714.30) also includes children with polyarticular JIA and unspecified JIA, charts were initially screened for specific diagnosis of sJIA. Children were included in the cohort if they were diagnosed with sJIA during the study period, meeting the International League of Associations for Rheumatology (ILAR) criteria [5].

### Data abstraction

Clinical data were obtained via review of the electronic medical record (EMR) using a standardized tool. Variables included patient demographics; characteristics of initial presentation, including history, physical exam findings, and the results of laboratory studies at diagnosis; medication regimens; responses to therapy; and escalation of therapy. We adapted the Wallace criteria [6] to define clinically inactive disease (CID) as absence of arthritis, systemic features, and laboratory abnormalities, if labs were performed. Physician global assessment of disease activity was not consistently recorded in the EMR so this criterion was not included in our definition. MAS was defined by the PRINTO criteria (fever, ferritin >684 ng/dl, and any 2 of the following: platelet count <181 × 10^9^/L, aspartate aminotransferase (AST) > 48 U/L, triglycerides >156 mg/dl, fibrinogen <360 mg/dl [7].

Statistical analyses: Children were divided into 3 comparison groups for analyses:

1. Children who achieved CID with NSAID monotherapy,
2. Children who received a trial of NSAID monotherapy at diagnosis but required additional medications, therefore failing NSAID monotherapy, and
3. Children who did not receive NSAID monotherapy at diagnosis

By definition, patients who received NSAID monotherapy did not receive any corticosteroids through their NSAID trial. If corticosteroids, DMARDs, or biologic medications were added to a patient’s regimen, it was considered a failure of NSAID monotherapy.

All analyses were conducted with SAS 9.4 (SAS Institute Inc., Cary, NC, USA). CRP at diagnosis was unknown for 6 of the 51 children in the cohort, and was imputed via multiple imputation using SAS Proc MI using the white blood cell count (WBC), hemoglobin (Hg), and platelet level.

In order to determine which patient demographic and disease presentation variables (initial joint count, presence of specific diagnostic criteria, lab values at diagnosis) were predictive of assignment of NSAID monotherapy as well as NSAID monotherapy success, we began by examining the bivariate relations between these variables and either the assignment to or response to NSAID monotherapy. Logistic regression was used for continuous variables and chi-square or Fisher’s exact test for categorical variables. Use of a single multivariate regression model was not practical given the high ratio of possible predictors to number of study participants. Continuous predictor variables were transformed into dichotomous variables to create clinically meaningful categories.

To create a multivariate model predictive of NSAID monotherapy response, we began with the patient demographic or disease presentation variable with the strongest bivariate association with NSAID monotherapy response and continued to add patient demographic or disease presentation variables in order of strength of bivariate association with NSAID monotherapy response. Any *p*-values <0.25 were included in the multivariate model, based on the Wald test from logistic regression [8].

## Results

We identified 1717 potential subjects using the administrative query. Of those, 87 children were diagnosed with sJIA during the study period and met ILAR criteria for sJIA. All children included in the cohort had both systemic and articular features present. Fifty-one children received a trial of NSAID monotherapy and 13 of those (25.5%) achieved CID with NSAID monotherapy. Of the children who were given a trial of NSAID monotherapy, 35 (69%) were prescribed naproxen at a mean dose of 18 mg/kg/day, 12 (24%) were prescribed indomethacin at a mean dose of 2.5 mg/kg/day, 3 (6%) were prescribed sulindac at a mean dose of 10 mg/kg/day, and 1 (2%) was prescribed ibuprofen at an unknown dose.

Results from the tests of bivariate associations with both the assignment of NSAID monotherapy as well as the response to NSAID monotherapy are shown in Table 1. Patients with serositis at diagnosis were significantly less likely to receive a trial of NSAID monotherapy (*p* = 0.003). Other patient demographic or disease characteristics (such as initial joint count and rash) were not associated with whether a child received a trial of NSAID monotherapy.

**Table 1:** Correlation of demographic features and disease characteristics with response to NSAID monotherapy

	Responded to NSAID monotherapy	Failed NSAID monotherapy	Did not receive a trial of NSAID monotherapy	p-value comparing trial (columns 1 and 2) vs no trial (column 3)	p-value comparing responded to trial (column 1) vs. failed trial (column 2)
Patients	13	38	36		
Demographic features					
Male, *n* (%)	7 (54%)	15 (39%)	17 (47%)	0.71	0.52
Age at presentation, mean (SD)	4.8 (3.6)	7.1 (4.7)	6.1 (4.6)	0.68	0.10*
Age ≤ 8 years at presentation, *n* (%)	11 (85%)	21 (55%)	27 (75%)	0.23	0.10*
Disease characteristics					
Initial joint count, mean (SD)	1.8 (1.6)	5.7 (9.0)	4.7 (6.3)	0.98	0.01**
Joint count ≤5, *n* (%)	13 (100%)	29 (76%)	29 (81%)	0.83	0.09*
Characteristic rash, *n* (%)	13 (100%)	34 (89%)	33 (92%)	0.93	0.56
Generalized lymphadenopathy, *n* (%)	2 (15%)	6 (16%)	9 (25%)	0.28	1.00
Hepatosplenomegaly, *n* (%)	2 (15%)	7 (18%)	5 (14%)	0.64	1.00
Serositis, *n* (%)	0 (0%)	1 (3%)	8 (22%)	0.003**	1.00
CRP ≤13 mg/dL*, *n* (%)	12 (92%)	26 (68%)	17 (59%)	0.14*	0.14*
MAS at diagnosis, *n* (%)	0 (0%)	2 (5%)	7 (19%)	0.03**	1.00
MAS ever, *n* (%)	0 (0%)	6 (16%)	11 (31%)	0.03**	0.32
WBC (K/μL), mean (SD)	16.7 (±7.0)	16.4 (±7.0)	19.6 (±10.1)	0.15	0.89
Hg (g/dL), mean (SD)	10.4 (±0.9)	10.1 (±1.5)	9.9 (±1.6)	0.45	0.47
Platelets (K/μL), mean (SD)	492 (±193)	486 (±222)	519 (±248)	0.57	0.93
ESR (mm), mean (SD)	80 (±26)	77 (±26)	75 (±31)	0.69	0.81
Ferritin (ng/mL), mean (SD)	410 (±722)	3042 (±4813)	3060 (±3777)	0.55	0.07*
Outcomes					
Days to clinically inactive disease, median (range)	49 (28-356)	764 (13-3806)	267 (0-2716)	0.60	0.0001**
Days to medication escalation, median (range)	n/a	32 (2-146)	n/a	n/a	n/a
Days to first follow-up, median (range)	48 (28-252)	21 (6-327)	27 (9-188)	0.33	0.004**
Days of disease duration prior to diagnosis, median (range)	55 (26-492)	46 (14-484)	81 (11-848)	0.25	0.60
Days of total follow-up, median (range)	1000 (154-3164)	1693 (0-4229)	956 (188-4678)	0.45	0.37

P-values below 0.25 have one asterisk (*) and were considered trending (based on Wald test from logistic regression). *P*-values below 0.05 have two asterisks (**) and were considered statistically significant. All *p*-values below 0.25 are bolded

When evaluating disease characteristics of patients who achieved CID with NSAID monotherapy, initial joint count was the only statistically significant predictor (*p* = 0.01). Age at presentation, ferritin, and CRP were trending towards significance (*p* = 0.10, *p* = 0.08, *p* = 0.14 respectively).

A multivariate model combined the three most significant/suggestive factors: age at presentation, initial joint count, and CRP at diagnosis. Ferritin was not included since it was unavailable in 45% of patients. In combination within the same patient, these three variables yield a very strong association with response to NSAID monotherapy (*p* = 0.002).

In the group of patients who failed NSAID monotherapy, 30 out of 37 patients (81%) achieved CID, while in the group of patients who did not receive NSAID monotherapy, 28 out of 34 (82%) patients achieved CID (*p* = 0.89). For those who failed NSAID monotherapy trial, median time to CID was 764 days, while for those who were not tried on NSAID monotherapy, the median time to CID was 267 days (*p* = 0.60). There was a significant difference within the group that received NSAID monotherapy, with the successful NSAID monotherapy group having a shorter time to CID than those who failed NSAID monotherapy (*p* = 0.0001). Within the group that failed NSAID monotherapy, the median time to drug escalation was 32 days. Regarding time to first follow-up, there was a significant difference, with patients successful on NSAID monotherapy taking longer to follow-up than those who failed NSAID monotherapy (*p* = 0.004). There was no difference in time to first follow-up visit between the group that received a trial of NSAID monotherapy versus not. There was also no difference between the groups with regard to disease duration prior to diagnosis or total follow-up time. Regarding the patient with 0 days to remission, that patient had a delay in presentation to a pediatric rheumatologist, and so was diagnosed retrospectively after symptoms had already resolved.

Two of the 51 patients who received NSAID monotherapy met criteria for MAS at diagnosis. One was 11 years old and had a CRP of 20.2 mg/dL at diagnosis. The second patient was 15 years old, with arthritis in 10 joints, and with a CRP of 7.4 mg/dL at diagnosis. Both patients’ regimens were escalated to include steroids and methotrexate within 2 weeks, and anakinra was added 1 month later with excellent response in both. The remaining children who developed MAS after diagnosis had already been escalated to corticosteroids, DMARDs, and/or biologics at the time their MAS developed.

Anakinra became widely used as a treatment for sJIA in 2007. Prior to 2007, 30 of 39 patients (77%) received NSAID monotherapy trial. After 2007, 21 of 41 patients (51%) received NSAID monotherapy trial. This difference is statistically significant (*p* = 0.02).

## Discussion

NSAID monotherapy is able to achieve CID in a small subset of children with sJIA. This study helps pediatric rheumatologists risk-stratify their patients at diagnosis when choosing whether to pursue a trial of NSAID monotherapy. Factors associated with achievement of CID with NSAID monotherapy include age ≤ 8 years at presentation, joint count ≤5, and CRP ≤ 13 mg/dL. When combined in a multivariate model, having all of these characteristics in the same patient have a very strong association with response to NSAID monotherapy (*p* = 0.002). Ferritin was also trending towards significance, but was unknown for 45% of patients who received NSAID monotherapy trial, so was not included in the multivariate model.

Conversely, children in our cohort who were older at diagnosis (> 8 years old), with more joints involved (>5), or with a higher CRP at diagnosis (>13), were less likely to respond favorably to NSAID monotherapy. When these variables are taken individually, joint count was independently statistically significant while age and CRP were trending towards significance. We recommend against NSAID monotherapy in a patient with sJIA with any of these unfavorable characteristics. In addition, we found that providers are less likely to trial NSAID monotherapy in the presence of serositis or MAS, and we agree with avoiding NSAID monotherapy in these groups, as these are indicators of more severe disease.

Of note, our study cohort spans the years 2000-2014, and anakinra became widely used as a first-line agent to treat sJIA in 2007. The introduction of anakinra did alter prescribing patterns at our institution. Prior to the anakinra era, 77% of patients received a trial of NSAID monotherapy. After 2007, this dropped to 51% of patients receiving a trial of NSAID monotherapy. This is consistent with our institutional practice becoming more aggressive with the initial treatment of sJIA.

There was no statistically significant difference in whether a patient achieved CID based on whether they received a trial of NSAID monotherapy or not. There was also no statistically significant difference in the time to CID between the group that received a trial of NSAID monotherapy and the group that did not. Despite this lack of statistical significance, the difference between 9 months versus 2 years of active disease is clinically significant for patients, their families, and their treating physicians. This leads us to the conclusion that if indicated, NSAID monotherapy trial should be very short, and if desired results are not achieved quickly, escalation should be aggressive. The group that is predisposed to achieve CID with NSAID monotherapy does so quickly (median time to CID was 49 days), and so if a significant response is not achieved within 2-4 weeks, medications should be transitioned to alternative first- and second-line agents, including corticosteroids. This adheres to the current ACR guidelines (2). In our cohort, there was no difference in time to first follow-up between patients who received versus did not receive a trial of NSAID monotherapy. However, there was a difference in time to follow-up between patients who achieved CID on NSAID monotherapy versus those who did not achieve CID, with those failing NSAID monotherapy having sooner follow-up. Therefore, all patients should receive a standard 2-4 week follow-up after initial diagnosis to determine whether medication regimen requires escalation; this recommendation is also consistent with the current ACR guidelines [2].

The two patients with MAS at diagnosis who received NSAID monotherapy had presenting characteristics (older age, high joint count, and elevated CRP) which were associated with poor response to this treatment (in addition to their presence of MAS). In retrospect, they should have initially been treated more aggressively. Fortunately, their medication regimens were quickly escalated and their disease came under good control within 6 weeks. The remainder of the patients in the NSAID monotherapy group who developed MAS did so after their medications had already been escalated to corticosteroids, DMARDs, and/or biologics. As previously stated, children with MAS either at presentation or at any point during their disease are not candidates for NSAID monotherapy.

This study has several limitations. The cohort was of modest size, so the number of characteristics that could be considered for analysis was limited. Selection bias was present in that providers chose to pursue NSAID monotherapy in only 51 out of 87 children diagnosed with sJIA during the study period. Disease activity scores could not be generated due to lack of patient global assessment in the majority of electronic medical records. Since this is an observational study, it is subject to the risk of unidentified confounders as well. CRP was not obtained for all patients at diagnosis, so the value was imputed for some subjects. ESR and ferritin were available for an even smaller number of patients at diagnosis, so these values could not be included in the multivariate model. Despite these limitations, this study represents an important first step in beginning to stratify initial therapy for sJIA based on clinical presentation. It will be critical to validate our findings in a larger cohort of patients, ideally in the setting of a clinical trial.

## Conclusions

- We retrospectively evaluated NSAID monotherapy in children with systemic juvenile idiopathic arthritis (sJIA), and examined patient characteristics to determine which aspects at diagnosis were associated with achievement of clinically inactive disease (CID).
- We found that NSAID monotherapy *should not* be considered in children with sJIA who are >8 years old at diagnosis, with >5 joints involved, or with an initial C-reactive protein (CRP) > 13 mg/dL based on our data. We also do not recommend NSAID monotherapy trial in children with serositis or MAS at diagnosis.
- If NSAID monotherapy trial is pursued, patients should follow up within 2-4 weeks to evaluate whether escalation of therapy is warranted.

## Abbreviations

AST: Aspartate aminotransferase
CARRA: Childhood arthritis and rheumatology research alliance
CID: Clinically inactive disease
CRP: C-reactive protein
CTPs: Consensus treatment protocols
DMARDs: Disease-modifying anti-rheumatic drugs
EMR: Electronic medical record
ESR: Erythrocyte sedimentation rate
Hg: Hemoglobin
ICD-9: International statistical classification of diseases and related health problems – 9
IL-1: Interleukin-1
IL-6: Interleukin-6
ILAR: International league of associations for rheumatology
JIA: Juvenile idiopathic arthritis
MAS: Macrophage activation syndrome
NSAIDs: Non-steroidal anti-inflammatory drugs
PRINTO: Pediatric rheumatology international trials organization
sJIA: Systemic juvenile idiopathic arthritis
WBC: White blood count
